# Effectiveness of ICT-based intimate partner violence interventions: a systematic review

**DOI:** 10.1186/s12889-020-09408-8

**Published:** 2020-09-07

**Authors:** Christo El Morr, Manpreet Layal

**Affiliations:** 1 School of Health Policy and Management, York University, 4700 Keele St, Toronto, Ontario Canada; 2 Global Health Program, York University, 4700 Keele St, Toronto, Ontario Canada

**Keywords:** Women, Intimate Partner Violence (IPV), Information Communication Technology (ICT), Virtual communities, Public health

## Abstract

**Background:**

Intimate Partner Violence is a “global pandemic”. Meanwhile, information and communication technologies (ICT), such as the internet, mobile phones, and smartphones, are spreading worldwide, including in low- and middle-income countries. We reviewed the available evidence on the use of ICT-based interventions to address intimate partner violence (IPV), evaluating the effectiveness, acceptability, and suitability of ICT for addressing different aspects of the problem (e.g., awareness, screening, prevention, treatment, mental health).

**Methods:**

We conducted a systematic review, following PRISMA guidelines, using the following databases: PubMed, PsycINFO, and Web of Science. Key search terms included women, violence, domestic violence, intimate partner violence, information, communication technology, ICT, technology, email, mobile, phone, digital, ehealth, web, computer, online, and computerized. Only articles written in English were included.

**Results:**

Twenty-five studies addressing screening and disclosure, IPV prevention, ICT suitability, support and women’s mental health were identified. The evidence reviewed suggests that ICT-based interventions were effective mainly in screening, disclosure, and prevention. However, there is a lack of homogeneity among the studies’ outcome measurements and the sample sizes, the control groups used (if any), the type of interventions, and the study recruitment space. Questions addressing safety, equity, and the unintended consequences of the use of ICT in IPV programming are virtually non-existent.

**Conclusions:**

There is a clear need to develop women-centered ICT design when programming for IPV. Our study showed only one study that formally addressed software usability. The need for more research to address safety, equity, and the unintended consequences of the use of ICT in IPV programming is paramount. Studies addressing long term effects are also needed.

## Background

Intimate partner violence includes physical violence, sexual violence, stalking, and psychological harm inflicted by a current or former partner or spouse [1]. Violence against women (VAW) has been described as a “global pandemic” by the United Nations [2]. It is considered both a violation of women’s human rights [3] and a public health issue [4]. In low- and middle-income countries, violence against women is widespread and often involves pregnant women [5, 6]. However, violence against women occurs in high-income countries as well [7, 8]. Nearly one in three women have experienced intimate partner violence or sexual violence [9]; therefore, it is important to disseminate as widely as possible the knowledge and tools related to IPV prevention and to intervention to empower the women subjected to IPV. Information and communication technologies (ICT) present an opportunity for such dissemination. ICT are being adopted at unprecedented rates in high-income as well as low- and middle-income countries [10]. Moreover, the use of the internet [11–37], mobile phones, and smartphones [36, 38–43] for health purposes has been well documented in research. It has been used to address chronic disease management [44, 45], mental health challenges [46, 47], and hospital readmissions [48], encompassing applications that target the public (i.e., public health informatics), interactions between patients and healthcare professionals, and applications for individual use through smartphone apps (i.e., consumer health informatics). However, little is known about the use of ICTs in the context of violence against women, and only a few articles on the subject have been published recently [43, 49, 50]. At the same time, there is a solid increase in phone ownership and access to the internet in low- and-middle-income countries [51], which suggests the possibility of implementing ICT-based interventions to address IPV in these countries.

Recent systematic reviews showed that the efficacy of ICT-based mobile apps for health (mHealth) is still limited, as research in the field lacks long-term studies and existing evidences of impact are inconsistent [52]. Also, mHealth in the domain of violence against women (VAW) showed an abundance of apps addressing one-time emergency or avoidance solutions, and a paucity of preventative apps, which indicates the need for studies addressing data security, personal safety, and efficacy of interventions using apps to address VAW [53]. By extension, investigating the situation of ICT in IPV seems a necessary step.

Given the existing IPV interventions challenges, the evidence demonstrating effectiveness of online interventions in health, the rise of research on online IPV interventions, the risks inherent in ICT use for IPV programming, it is important to synthesize the available evidence regarding the use of ICT-Based IPV interventions. To our knowledge, there is no systematic review of such work. To address this knowledge gap, we initiated a systematic review of literature on ICT-Based IPV interventions. The study objectives were to examine whether ICT could become acceptable for effective IPV interventions, we reviewed the literature on the use of ICT-based interventions to address IPV issues. The questions that guided us in examining the were as follows: (1) “what type of objectives did ICT based interventions tried to address?”, (2) “were ICT based interventions effective in addressing IPV?”, and (3) “what type of strategies did they implement to mitigate ICT risks (e.g. safety, data security)”. The results will inform future ICT-based IPV interventions.

## Methods

A systematic review was conducted, employing a digital search of bibliographic databases: PubMed, PsycINFO, and Web of Science. The literature was systematically screened by titles and abstracts and by applying key search terms. The following search terms were used: women, violence, domestic violence, intimate partner violence, information, communication technology, ICT, technology, email, mobile, phone, digital, ehealth, web, computer, online, and computerized. The full list of search terms is provided in Table 3 (See Appendix). Studies were included if they described an intervention that used some form of ICT, and if the recipients were women who experienced intimate partner violence or domestic violence, no matter what was the intervention type, comparison group, outcomes, study design, who was providing the intervention. We excluded studies that did not focus on ICT, studies where interventions were not aimed at women with IPV experiences, studies that described protocols, were not written in English, or were not full text, as well as journal articles and chapters in books. Non-English-language articles were excluded because no evidence exists of systematic bias caused by language restrictions [54]. The literature search was not subjected to any time limitations. The most recent search was completed on June 30, 2020.

The literature search, review, and data collection from articles was conducted by a single individual and was repeated by one other individual, the two resulting articles were then integrated. A meta-analysis was not conducted because of the disparities in study design, variables, and exposures between the studies.

## Results

### Summary

**Fig. 1:**
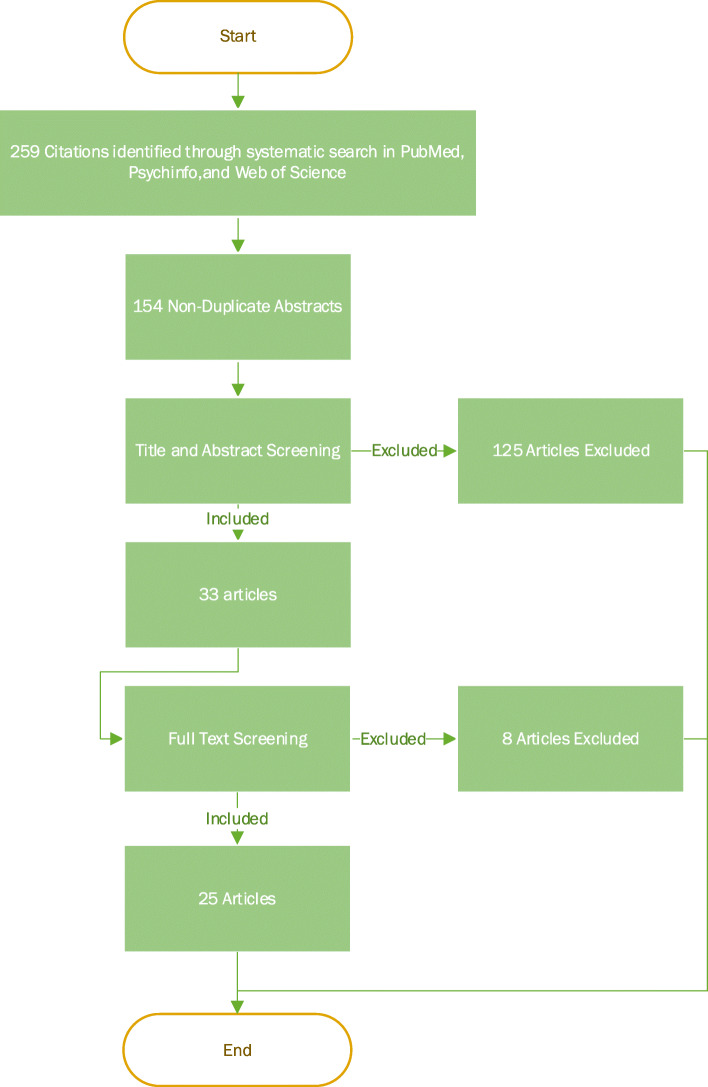
Flow chart for article identification and selection

In total, 259 articles were identified, among which 105 articles were duplicates. Out of the 154 unique articles, 125 were excluded based on the content of their abstracts. The inclusion criteria were then applied to the remaining 33 articles after reading their full text. Four articles were then excluded, and 25 articles were kept for analysis [9, 55–78] (Fig. 1).

Table 4 (see Appendix) lays out the studies in terms of population, intervention, comparison groups, and outcomes (PICO). Table 1 presents the authors, publication year, study country, study type, recruitment space, theme, outcomes, sample size, sample size per arm, control group, and the type of ICT Used for the 25 studies. Out of the 25, 23 (92%) took place in North America (20 studies (80%) in the United States and 3 (12%) in Canada), 1 study (4%) took place in Australia, and 1 (4%) in New Zealand.

**Table 1:** Summary of the 25 studies

Author	Year	Country	Study Type	Recruitment Space	Theme	Outcomes	Sample Size	Sample Size per Arm	Control group	ICT Used
Ahmad. F [55].	2009	Canada	RCT (2 arms)	Medical services	Screening and Disclosure	Count	293	146.5	Usual care (no online screening)	Desktop/Laptop
Bacchus. L.J. et al. [56]	2016	USA	Cross Sectional	Community wide	Screening and Disclosure	Count	28	28	Face-to-Face Paper based screening	Tablet
Braithwaite SR and Fincham FD [57]	2014	USA	RCT (2 arms)	Community wide	IPV Prevention	CTS2	52	26	Static information and HomeWorks	Desktop/Laptop
Chang. J. C. et al. (2012) [58]	2012	USA	Pre-post	Medical services	Screening and Disclosure	NVQ	50	50	Same as Intervention Group; audio recorded their first visits to the provider	Desktop/Laptop
Choo E. K. et al. [59]	2016	USA	RCT (2 arms)	Social services	ICT Suitability	CSQ-8, SUS	40	20	Same website with an irrelevant content (fire safety) + phone booster	Tablet + Phone
Constantino. R. E. et al. [60]	2015	USA	RCT (3 arms)	Social services	Screening and Disclosure	IPVEQ, PRQ, ISEL, PROMIS	32	11	Arm2: Face-to-face screening: same material Arm3: ARM #3 = Waitlist/Control	Desktop/Laptop
Eden. K. B. et al. [61]	2015	USA	RCT (2 arms)	Community wide	Support, Decisional conflict	DCS, DA/DA-R	708	354	Standard safety planning online information + Resource website	Desktop/Laptop
Fincher D. (2015) [62]	2015	USA	RCT (2 arms)	Medical services	IPV Prevention	CTS2	368	184	Face-to-face interview	Tablet
Fiorillo. D. et al. [63]	2017	USA	Pre-post	Medical services	Mental Health	LEC-5, SLESQ, DASS, PCL	25	25	Same as Intervention Group	Desktop/Laptop
Ford-Gilboe M et al. [64]	2020	Canada	RCT (2 arms)	Community wide	Mental Health	CESD-R, PCL-C	531	265.5	Static/Standard Non-tailored version of the same interactive website	Unknown
Gilbert. L. et al. [65]	2016	USA	RCT (3 arms)	Legal services	IPV Prevention	CTS2	306	102	Arm2: 4 Face-to-face traditional group sessions: same material Arm3: 4 weekly sessions for wellness promotion	Desktop/Laptop
Glass. N., Eden.K. et al [66]	2010	USA	Pre-post	Social services	Support	DCS	90	90	Same as Intervention Group	Desktop/Laptop
Hassija C. and Gary MJ [67]	2011	USA	Pre-post	Medical services	Mental Health	PCL, CESD	15	15	Same as Intervention Group	Desktop/Laptop
Hegarty K et al. [68]	2019	Australia	RCT (2 arms)	Community wide	Support, Mental Health	GSE, CESD-R	422	211	Static intimate partner violence information	Unknown
Humphreys. J. et al. [69]	2011	USA	RCT (2 arms)	Medical services	Screening and Disclosure	Count, AAS	50	25	Usual care (no online screening)	Desktop/Laptop
Koziol-McLain. J. et al [9]	2018	New Zealand	RCT (2 arms)	Community wide	IPV Prevention	CESD-R, SVAWS	412	206	Static/Standard Non-individualized web-based information	Desktop/Laptop
MacMillan. H.L. et al. [70]	2006	Canada	RCT (3 arms)	Medical services	IPV Prevention	PVS, WAST, CAS	2416	805	Arm2: Face-to-face interview Arm3: written self-completed questionnaire	Desktop/Laptop
McNutt L. A.et al. [71]	2005	USA	RCT (3 arms)	Medical services	Screening and Disclosure	Count	211	70	Arm2: Face-to-face screening with a nurse: same material (Short questionnaire) Arm3: Computer screening (Long questionnaire)	Unknown
Renker, P. R., & Tonkin, P [72].	2007	USA	Cross Sectional	Medical services	Screening and Disclosure	NVQ	519	519	No Control Group	Desktop/Laptop
Rhodes et al. [74]	2002	USA	RCT (2 arms)	Medical services	Screening and Disclosure IPV Prevention	PVS, AAS	470	235	Usual care (no online screening)	Desktop/Laptop
Rhodes. K.V. et al. [73]	2006	USA	RCT (2 arms)	Medical services	Screening and Disclosure IPV Prevention	PVS, AAS	1281	640.5	Usual care (no online screening)	Desktop/Laptop
Scribano et al. [75]	2011	USA	Prospective	Medical services	Screening and Disclosure	Count	13,057	13,057	Face-to-Face screening	Kisok
Sprecher. A. G. et al. [76]	2004	USA	Diagnostic Case-Control (AI)	Medical services	Screening and Disclosure	AI	19,830	19,830	No control group	Desktop/Laptop
Thomas. C.R. et al. [77]	2005	USA	Prospective	Social services	Mental Health	SCL-90-R	35	35	No control group	Desktop/Laptop and Telephone
Trautman. D. E. et al. [78]	2007	USA	RCT (2 arms)	Medical services	Screening and Disclosure	Count	1005	502.5	Usual care (no online screening)	Desktop/Laptop

Most studies focused on women with potential vulnerability to, past experience of, and/or current experience of intimate partner violence, with the exception of one [74], which included both men and women as study participants. Four studies included women who were pregnant [56, 58, 69, 72]; two of these studies included women up to 3 months postpartum who had history of IPV [56, 72]. Two studies focused on women with a history of IPV and who were active substance(s) users [59, 65], and 1 study on women who were at risk of HIV through unprotected intercourse [65].

Out of the 25, 17 studies (68%) were solely desktop- or laptop-based [9, 55, 57, 58, 60, 61, 63, 65–67, 69, 70, 72–74, 76–78], 2 studies (8%) were solely tablet-based [56, 62], 1 study (4%) used computer and telephone [77], 1 study (4%) used tablet and telephone [59], 1 (4%) implemented a kiosk system [75] and 3 (12%) were not reported and supposed any type of ICT [64, 68, 71].

### Studies’ designs and interventions

Table 2 shows the characteristics of the included studies. The 25 studies included 16 randomized controlled trials (12 two-arm and four three-arm studies), four pre-post designs, two cross-sectional studies, two prospective studies, and one diagnostic case-control study (i.e. retrospective data with known disease-positive and disease-negative cases [79]).

**Table 2:** Characteristics of the included studies

Characteristics	# of studies
	(***N*** = 25)
**Country:**	
United states	20
Canada	3
^a^ Other	2
**Main focus**	
Screening and Disclosure	13
IPV Prevention	5
Treatment (Mental Health)	4
Empowerment /Support	2
ICT Suitability	1
**Recruitment Space**	
Medical Services	14
Community wide	6
Social Services	4
Legal Services	1
**Sample size:**	
RCT (2 arms)	40 to 1281
RCT (3 arms)	32 to 2416
Pre-Post	15 to 90
Cross-Sectional	28 to 519
Prospective	35 to 19,830
Diagnostic Case-Control	13,057
**Type of Study**	
RCT (2 arms)	12
RCT (3 arms)	4
Pre-Post (one arm)	4
Cross-Sectional	2
Prospective	2
Diagnostic Case-Control	1
**Study Settings**	
Urban	14
Suburban	2
Mixed	3
Setting Not reported	6

^a^ Austria and New Zealand

Control groups varied widely, and wait-list controls were used in five RCT studies [55, 69, 73, 74, 78]. Four studies allowed control groups to access websites with static, or non-interactive, or non-tailored content [9, 57, 64, 68], while two studies used irrelevant information for control groups [59, 61], seven control groups used face-to-face (or paper-based self-reported) screening [56, 60, 62, 65, 70, 71, 75], four had the intervention group play the role of control (i.e. pre-post design) [58, 63, 66, 67], and three studies had no control groups [72, 76, 77]. The sample size in the RCT studies varied extensively from 32 participants to a high of 2416.

The 25 interventions implemented had various foci. ICT was used for screening and disclosure in 13 (52%) of the studies. Five studies (20%) aimed at IPV prevention, four (16%) studies used ICT to address the mental health of female victims of IPV, and two (8%) studies used ICT to provide support for decision aid. Only one (4%) study assessed mainly the suitability of ICT for use in an IPV context.

The 25 studies had five types of interventions and varied study settings. In terms of settings, 14 studies were conducted in medical services facilities [55, 58, 62, 63, 67, 69–76, 78] such as emergency departments, clinics, community health centers, trauma treatment centers, and family practices. Six studies were conducted in the community [9, 56, 57, 61, 64, 68], four in social services facilities [59, 60, 66, 77], and one in legal services facilities [65].

The 25 studies represent a range of uses of ICT in the context of IPV, addressing screening and disclosure, IPV prevention, ICT suitability, empowerment and support, and women’s health.

#### Screening and disclosure

In three studies, IPV screening using ICT was found to be as effective as using the usual face-to-face/paper method [58, 70, 71]. One study reported that computerized screening was more sensitive and less or similarly specific compared to face-to-face staff screening [71]. One study reported high self-disclosure of IPV using computers vs in person IPV screening with health professionals; out of 250 female patients who participated in both screening methods. 67(27%) patients out of the 250 disclosed some form of IPV in person compared to 85 (34%) who disclosed IPV via a computer. Out of those 85 patients, 60 (71%) also disclosed IPV to their doctors in person and 24 patients (26%) disclosed via a computerized tool but not with the doctor [58].

One study that included African American women in a women, infants, and children (WIC) services setting found that women were less likely to disclose IPV using a computerized intervention than in person [62]. A study that used a tablet for disclosure during perinatal home visitation found the tablet to be a conduit through which interpersonal connection between women and home visitors was facilitated [56]. One study found that women were more likely to disclose IPV using ICT, leading to higher rates of screening and disclosure [78]. One study reported that 81.8% of women disclosed using the ICT intervention, and only 16.7% women disclosed using usual care [69]. Another study found that implementing ICT-based disclosure in an emergency department was successful and reliable [75].

#### IPV prevention

Two studies addressed IPV prevention [57, 65]. One study showed that 62% of the participating women who used ICT were less likely to report experiencing physical IPV at a follow-up (12 months later), 76% were less likely to report IPV with injury, and 78% were less likely to report severe sexual IPV [65]. The study by Braithwaite et al., which targeted both males and females using ICT, reported less physical aggression committed by females at post-intervention, as well as less physical aggression committed by both males and females at a 1-year follow up; also, the study showed a large reduction in expected counts for female- and male-perpetrated physical aggression at the 1-year follow-up (71 and 99%, respectively) [57].

#### Women’s health

Our systematic review showed that ICT has been used to address two aspects in the lives of some women experiencing IPV: substance use and mental health. Six studies used online tools to address the mental health of women experiencing IPV [9, 55, 60, 63, 67, 77]. Depression was measured in five studies [9, 55, 60, 63, 67], anxiety was measured in three [60, 63, 77] and stress in two [63, 67, 77]. In all studies, mental health showed improvement compared to intervention. One study reported that women found it easier and safer to report drug use and partner abuse through a computer than in person [77]. The study by Hassija et al. addressed the treatment of IPV-related trauma through video conferencing, and found the method effective at reducing post-traumatic stress disorder (PTSD) symptoms, with high users’ satisfaction [67].

#### Empowerment and support

ICT was used to empower women by enabling them to create safety and action plans and by providing them with tools for enhanced decision making and self-efficacy. Three studies focused on women creating a safety and/or action plan in the event of a future partner abuse incident [61, 66, 69], with two interventions providing additional local resources [61, 69]. In one study, 90% of the participating women who used ICT reported leaving their abusive partner within the year [66], and in another study 64% of the participating women reported the intention to make changes in regard to their IPV within 30 days to 6 months [69]. Moreover, in a single study focused on using online tools to teach participants about behaviours and/or actions related to safety [9], researchers reported a 12% significant increase in safety behaviours for the ICT-based intervention group, compared to a 9% increase for the control waitlist [9]. In addition, a study reported that participants found using a computer survey to disclose IPV safer than a face-to-face survey [55].

In terms of decision-making and self-efficacy, two studies reported that more than 78% of the participants acquired general skills through the ICT-based interventions [9], and two other studies reported that participants gained decision-making skills through the ICT-based interventions [61, 66]. Additionally, using their new skills, women experienced lower decisional conflicts and had an overall less difficult time deciding on their actions [61, 66].

#### ICT suitability

Only 1 study has a formal testing for the usability of ICT software as a major focus using the Systems Usability Scale [59]. The results indicate high satisfaction with the software usability.

### Measurements

Table 5 (Appendix) summarizes the outcomes measured by each study. Our review revealed a wide variation among studies in terms of outcomes measured for studies that address the same focus. In total, 27 measurement tools were used in the 25 studies (see Table 5 in Appendix).

Among the 12 studies that address screening and disclosure, five studies used a simple disclosure count [55, 56, 69, 75, 78]. Two studies used non-validated questionnaires [58, 72], and two studies used the Partner Violence Screen (PVS) and the Abuse Assessment Screen (AAS) [73, 74]. Three studies had no common outcome measurement tools.

The four studies [63, 64, 67, 77] that focus on mental health used eight different outcome measurement tools; only the PTSD Checklist (PCL) was common to two studies [63, 67].

In terms of suitability of ICT, the Systems Usability Scale (SUS) was used in one study only to assess software usability [59].

Out of the five studies [9, 57, 62, 65, 70] focusing on IPV prevention, three studies [57, 62, 65] used the Revised Conflict Tactics Scale (CTS2). The two studies that addressed support [66, 68] had no common measurement tools.

## Discussion

### Principal findings

Our review revealed the emerging nature of ICT use in IPV research. While there is a growing interest in the use of ICT in IPV interventions, there are virtually no studies examining its challenges.

While most of the studies used ICT to enhance screening and increase the disclosure rate, few studies targeted IPV prevention and even fewer aimed at improving support. Suitability of ICT was seldom assessed in a formal way using a validated usability scale (e.g. Systems Usability Scale) or methodology [80].

In addition, while most of the studies used RCT design, the number of arms, the population, the control groups used, the sample sizes, and the outcome measures varied widely among the studies, which makes it hard to compare those results. With the exception of two large sample sizes that were used in two non-RCT studies (one that accessed electronic health records for an artificial intelligence application [76], and another that used the emergency department [75]), the sample sizes per arm were generally low. The sample size per arm was less than 30 in four studies. Only six studies had a sample size per arm between 100 and 300, and only four studies had a sample size per arm between 300 and 805. This suggests that the current ICT-based IPV interventions have limited generalizability and comparability—especially because only six studies were conducted in the community.

Twenty-three (92%) of the studies were conducted in North America, 20 (80%) of which were in the United States, which is an additional limitation to the generalizability of the findings since they lack diversity in terms of ethnicity, race, language, and cultural backgrounds. Diversity is crucial in IPV. Research shows that foreign-born immigrant as well as indigenous women are more likely to experience IPV [81, 82] and intimate partner homicide than other women [83, 84]; hence, addressing diversity in IPV is critical. It is encouraging that one recently published RCT protocol laid out a plan for culturally tailored intervention targeting immigrant, refugee, and indigenous survivors of IPV [85].

### Equity

Technology is costly in terms of hardware, software and data plan costs. Consequently, while access to ICT by women experiencing IPV is a challenge in high income countries, including the United States [86, 87], it is even more difficult in low- and middle-income countries (LMICs). This creates inequity in access to technology, and a digital divide among women subject to IPV. This inequity challenge and its impact on outcomes has long been observed in electronic health (eHealth) [88, 89] and needs to be addressed in ICT-based IPV interventions; it was not addressed in the studies covered by our review. Also, involvement of users in software design is a well-known need that is effective in producing software that works for users and aligns with their priorities and is suitable for their environments [47, 90–94]. Hence, involving women experiencing IPV in the research team and in the ICT software design process is paramount to ensure usability and accessibility of the software and as a matter of equity [95]. There is a lack of research in this area in the studies covered by our review.

A recent study protocol is promising that ICT will ensure lower access barriers [96], which is the traditional unchecked point of view; this is another demonstration of the need to shed a critical light on the use of ICT for women experiencing IPV, analyzing equity as well as the safety and ethical challenges involved.

### Safety and ethical challenges

Our review shows that 8 studies [55, 56, 59, 63, 64, 70, 72, 75] reported that women found ICT interventions suitable for IPV disclosure; three of those studies found it particularly suitable in terms of confidentiality, usefulness, and satisfaction [56, 63, 72]. Stigma is an important factor associated with intimate partner violence [97] limiting agency in help-seeking for IPV [98]; ICT seems to be a tool that provide an opportunity for women subject to IPV. With the exception of one in which participants preferred a face-to-face discussion [62], IPV disclosure through ICT was found to be most appropriate in most of the studies compared to face-to-face disclosure and was perceived as non-judgemental and more anonymous than face-to-face discussion, which facilitated more disclosure.

The increase in phone ownership and internet access in low- and middle-income countries [51], coupled with the ability to use ICT to target individuals through health informatics tools that targets individuals (i.e. consumer health informatics) [99] such as apps, makes ICT a flexible tool to address IPV in multiple languages, embedding different cultural cues, and overcoming the cultural stigma related to disclosing IPV from the convenience of a personal ICT device (e.g. cell phone, smart phone). Simple ICT tools such as cell phones are available in rural areas and proved to be successful tools in the health domain (e.g. chronic disease management) [100–102]. However, it is important to note that one challenge of ICT-based interventions is that only women with basic literacy and IT knowledge can benefit; also, some victims may not have access to ICT, and some abusers may restrict their partners’ access to ICT. Therefore, in addition to the traditional security considerations related to the use of ICT, such as maintenance of privacy [103] and confidentiality [99], there are ethical issues related to the unintended consequences of ICT [104, 105], including safety risks.

In the IPV domain, sharing cell/smart phones at home or with neighbors is a common practice [106, 107], which might increase the risk of IPV if the perpetrators notice that women are using these devices to address IPV [108]. The studies covered by this review were located in high income countries; there is little to no examination of the problem of access to ICT (i.e. cost), nor of the risks inherent in the use of ICT (e.g. sharing devices, ability to access browsing history) in addressing IPV programming in a variety of contexts. Ethical challenges related to the safety of women increase when women are sharing cell/smart phones with perpetrators; in such contexts special considerations should be taken care of, including “safety by design” [109].

Safety challenges involved in the use of ICT in health have recently attracted much attention [105, 110]. Moreover, recent reflections related to the ethical challenges of using web-based RCT show the need to equip participants with information about Internet safety [111]; likewise, identifying and managing safety risks within ICT-based IPV research remains a perspective to be explored. This raises ethical questions related to the use of ICT, for example in the case of referral embedded in the IPV programming, as was the case in three studies included in this review [55, 73, 78]. Poor quality services are well documented in low-resource and rural areas [112], so referring women to such services might have negative consequences for them. While this is not an ICT issue, ICT facilitates communication of information and has the potential to exacerbate current challenges. This is part of the well-known unintended consequences of the use of information technology in health [113–115].

### Future directions

Of the 19 studies that explicitly mentioned their settings, 14 were in urban settings, only three were in urban and suburban areas, and two were in suburban settings, suggesting a need to test ICT use for IPV in rural settings [67, 77] and uncover any particularities compared to the urban context.

It is also worth noting that our systematic review has not included search terms regarding the user of ICT tools to address IPV for women with disabilities. However, in a quick assessment, when we searched in PubMed for research that addresses the use of ICT to address violence in the context of women with disabilities, our search revealed only two papers [116, 117]. The use of ICT to address IPV for this particular group of women is important to address in a separate study, as ICT accessibility may be challenging for women with certain types of disabilities, especially since there is evidence that IPV occurs at higher rates in this population compared to the general population [118–123], and that ICT can play a major role in empowering people with disabilities [124]. The use of ICT tools to address IPV for women with disabilities, and the accessibility of these tools, remains an important area for future studies.

Moreover, our review indicated that there is a paucity of research addressing ICT use for IPV prevention and IPV treatment. There is a clear need for more research on ICT-based interventions to prevent IPV and to address post-IPV challenges, such as mental illness and the integration and coordination of mental and social services (e.g., employment, housing), which has never been addressed in the reviewed literature. In this context, virtual communities may play an important role in integrating and coordinating mental health services and social services [125]. While the studies showed different aspects of ICT use for IPV, a more integrative approach can be taken if researchers approach IPV using a virtual community framework. A virtual community (VC) is defined as a community of individuals cooperating using online tools to attain an objective [126]. Health VCs have been used in healthcare to provide patients with education, health education, and remote support; that proved to be an enabling and empowering factor, which allowed patients to become active participants in managing their health conditions [127, 128]. Support was not provided solely by health professionals; instead, health VCs connected individuals with common experiences (e.g., similar health conditions), which enabled them to interact and mutually support each other [129]. Healthcare providers could provide validated evidence-based health information, coupled with strategies for effective chronic disease management [130–132]. Ample evidence exists demonstrating that virtual tools are effective and efficient for addressing health issues experienced by patients with various health conditions (chronic kidney disease, pulmonary hypertension, cancer) [126, 132–134]. There is also ample evidence that health VCs are effective in engaging individuals managing their own health condition [131, 132]. Moreover, VCs can be patient-centred, customizable to individual preferences, and responsive to individuals’ needs and values [135]. In terms of mental health, an important factor for women experiencing IPV, VCs provide a secure, private way for women to communicate privately and securely and to access information tailored to their situation in a personalized manner. This privacy facilitates access and assists in overcoming stigma, especially for women from visible minority groups [136]. VCs have a proven potential to engage participants [137]. There is ample evidence that health VCs are associated with positive mental and social benefits, such as reduced loneliness and increased emotional well-being, self-esteem, and self-empowerment [129, 138, 139]. It is important to explore an integrative approach to ICT-based intervention in IPV using VCs, especially since VCs enable a community dimension that facilitates mutual support and empowerment among its members (e.g., abused women).

### ICT vs. paper

In a study that screened for IPV, while women preferred computerized over face-to-face disclosure, computerized screening did not increase prevalence, so ICT did not lead to increase in disclosure. Also, when women disclosed by answering paper-based questionnaires, the self-completed paper-based questionnaires had less missing data collected than both computer-based and face-to-face interviews [70], which shows the advantage of having for paper-based screening (i.e. less missing data).

Likewise, while ICT allowed considerably higher IPV detection, this did not always lead to charting for IPV or to a follow-up by treating physicians [74]; more research is needed to understand the factors, such as continuing medical education [140], that increase the chances of charting and follow-up. Detection is not enough.

We have noted above the lack of research regarding equity, safety, and the ethical challenges involved in the use of ICT, as well as the lack of culturally, ethically, and racially sensitive ICT programs. ICT might be able to support and enhance more traditional on-the-ground program delivery; however, ensuring that effective ICT-based interventions reach the most vulnerable in equitable, ethical, and safe ways remains a research agenda to be undertaken.

Current results suggest that face-to-face and paper-based approaches should not be discarded, and that the computer-based software design must be user-centred and must follow usability principles [141, 142].

### Limitations

Limiting the search to English language is one of the limitations of this study. Another limitation was the difficulty to compare the results, since the tools used to measure the same outcome varied widely between the studies. Various questionnaires were used to detect IPV, assess decisional conflict, assess mental health challenges, assess treatment efficacy, and assess different primary and secondary outcomes. An illustrative example is the varied questionnaires that researchers used to measure IPV [55, 57, 60, 62, 65, 69, 73–75], which included the use of an artificial neural network to identify IPV automatically via analysis of the notes stored in the electronic health records [76]. Our review shows that there are limits for comparing the effectiveness of the interventions in terms of mental health (e.g., reduction in stress, anxiety, or depression levels), given the great variety of mental-health-related measurement tools that have been used.

## Conclusion

The evidence reviewed suggests that ICT-based interventions have the potential to be effective in spreading awareness about and screening for IPV. ICT use show promise for reducing decisional conflict, improving knowledge and risk assessments, and motivating women to disclose, discuss, and leave their abusive relationships. However, there is lack of homogeneity among the studies’ outcome measurements, and the sample sizes, the control groups used (if any), the type of interventions and the study recruitment space.

The use of ICT-based interventions seems to be an attractive option for disseminating awareness and prevention information [143], due to the wide availability of ICT (including simple mobile phones) in both high-income and low- and middle-income countries. ICT may also present an opportunity to deliver culturally sensitive multilingual interventions using consumer health informatics. However, there is a clear need to develop women-centred ICT design when programming for IPV. Our study showed only one study that formally addressed software usability. Moreover, research directly addressing safety, equity, and ethical challenges in using ICT in IPV programming are virtually non-existent; the need to find answers to equity, and the unintended consequences of the use of ICT use for IPV programming is necessary. In this context, virtual communities may play an important role in providing a sense of community and in integrating and coordinating the services around women experiencing IPV. Future longitudinal follow-ups could help determine the long-term effects of the use of ICT in IPV programming.

## Data Availability

Data sharing not applicable to this article as no datasets were generated or analysed during the current study.

## Appendix

**Table 3:** Search Terms

**PubMed**	
1	(((women[Title]) AND violence[Title])) AND English[Language]
2	((domestic[Title]) AND violence[Title]) AND English[Language]
3	(Intimate Partner Violence[Title]) OR IPV[Title] AND English[Language]
4	((information and communication technology[Title]) OR ICT[Title] OR technology[Title] OR email[Title] OR mobile[Title] OR phone[Title] OR digital[Title] OR ehealth[Title] OR web[Title] OR computer[Title] OR online[Title] OR computerized[Title]) AND English[Language]
	(1 OR 2 OR 3) AND 4
**PsycINFO**	
1	ti(women) AND ti(violence) AND la.exact(“English”)
2	ti(Domestic Violence) AND la.exact(“English”)
3	ti(Intimate Partner Violence) OR ti(IPV) AND la.exact(“English”)
4	(ti(information AND communication technology) OR ti(ict) OR ti(technology) OR ti(email) OR ti(mobile) OR ti(phone) OR ti(digital) OR ti(ehealth) OR ti(web) OR ti(computer) OR ti(online) OR ti(computerized) AND la.exact(“English”))
5	(1 OR 2 OR 3) AND 4
**Web of Science**	
1	(ti = (women) AND ti = (violence)) AND LANGUAGE:(English)
2	(ti = (Domestic Violence)) AND LANGUAGE: (English)
3	(TI = (Intimate partner Violence OR IPV)) AND LANGUAGE: (English)
4	(TI = (information communication technology) OR TI = (ict) OR TI = (technology) OR TI = (email) OR TI = (mobile) OR TI = (phone) OR TI = (digital) OR TI = (ehealth) OR TI = (web) OR TI = (computer) OR TI = (online) OR TI = (computerized)) AND LANGUAGE: (English)
	(#1 OR #2 OR #3) AND #4

**Table 4:** PICO table

Author, Year	Population	Intervention (Study Design, Perspective, Time Horizon)	Comparison Group	Outcome (Results)
Ahmad. F., et al. (2009) [55].	Female patients -at least 18 years of age, -in a current or recent intimate relationship (within the last 12 months), -were able to read and write English **Setting**: Family practice clinic in an urban Hospital *N* = 293 women	**Intervention Type:** RCT (2 arms) **Intervention**: usual care + computer survey **Duration**: 7 months (March–September 2005) **Follow-up**: NA Intervention group: 144 **Primary Outcome** -Initiation of discussion about risk for IPVC (discussion opportunity) -detection of women at risk based on review of audiotaped medical visits. **Secondary outcomes** provider assessment of patient safety provision of appropriate referrals and advice for follow-up patient acceptance of the computerized screening **Measurement Tools** IPVC questions from: Abuse Assessment Screen, Partner Violence Screen, items from Improving Health Care Response to Domestic Violence: A Resource Manual For Health Care Providers Depression questions Center for Epidemiologic Studies Depression scale, Hamilton Rating Scale for Depression, Geriatric Depression Scale Computer Acceptance acceptance of computer-assisted screening by using the Computerized Lifestyle Assessment Scale (CLAS)	Control group: 149 Usual care	Attrition: 7% **Primary outcomes** -Computer screening was associated with statistically significantly more opportunities for discussing and detecting mental health disorders -Opportunity to discuss IPVC arose for 35% (48/139) in the computer-screened group and 24% (34/141) of the usual care group -Detection of IPVC occurred in 18% (25/139) of the computer-screened group and 9% (12/141) of the usual care group **Secondary outcomes** - provider assessment of patient safety: In IPVC positive detections, Physicians assessed patient safety more often in the computer-screened group: 9 of 25 participants in intervention vs 1 of 12 participants usual care group- Provision of appropriate referrals and advice for follow-up: 3 patients in the computer-screened group and 1 in the usual care group received referrals. During these visits, physicians asked patients to set up a follow-up appointment more often in the computer-screened group (20 of 25 participants) than in the usual care group (8 of 12 participants). - Patient acceptance of the computerized screening: Participants agreed that screening was beneficial but had some concerns about privacy and interference with physician interactions
Bacchus. L.J. et al. (2016) [56].	women aged 25 to 66 years pregnant or up to 3 months postpartum with prior IPV Setting: women enrolled in a US-based randomized controlled trial of the DOVE intervention *N* = 26 Women Interviewed (18 IPV positive)	**Intervention Type**: Cross Sectional (interviews) Intervention Group: 8 **Intervention**: tablet application Visitation Program (DOVE) to disclose IPV **Intervention Group**: 8 women (8 IPV positive and 1 IPV-negative) used the DOVE tablet application	Home Visitor paper-based Method *N* = 18 (11 IPV positive and 7 IPV negative)	−18 women were IPV positive - mixed feeling about the DOVE program (impediment vs facilitator) -patient-provider relationship is paramount - mHealth should be considered as a supplement and enhance therapeutic relationship - mHealth should be flexible and adapt to changing patient context
Braithwaite SR and Fincham FD (2014) [57]	Married Couples **Setting**: community 52 couples (*N* = 104)	**Study Type**: RCT (2 arms) **Intervention**: presentation, online videos, weekly home assignments, emails **Duration**: 6 weeks **Follow-up**: 1 year **Intervention Group: 25** **Outcome** IPV: measure by Revised Conflict Tactics Scale (CTS-2).	Active Control Group: 26 Presentation and inert information and HomeWorks	**Self-reported Physical aggression** receiving ePREP was associated with -less female-perpetrated physical aggression at post-treatment - less male-perpetrated physical aggression at 1-year follow up - and less female-perpetrated physical aggression at 1-year follow up - 71% reduction in expected counts for female-perpetrated physical aggression and a 99% reduction in expected counts of male perpetrated physical aggression at the 1-year follow-up **Partner-reported physical aggression** receiving ePREP was associated with - an increase in female-perpetrated physical aggression at post-treatment - significant decreases in female perpetrated physical aggression at the 1 year follow-up - 97% reduction in expected counts of physical aggression **Self-reported psychological-aggression** receiving ePREP was associated with a significant reduction in self-reported male-perpetrated psychological-aggression at the 1 year follow-up Gains were maintained at a 1-year follow-up assessment
Chang. J. C. et al. (2012) [58]	Women ages 18 years or older Pregnant English-speaking Coming for first OB/GYN visit **Setting**: hospital-based prenatal clinic *N* = 250 patient (for 50 providers)	**Intervention Type: cross-sectional Survey** **Intervention**: Computerized non-validated questionnaire **Duration**: NA **Follow-up:** 4 weeks after survey (Semi-structured interviews with those who reported experiencing IPV) **Intervention group:** 250 patients	Control group: Same as Intervention Group; audio recorded their first visits to the provider Control Size: 302 participants	Out of 250 women - 34% disclosed any type of IPV via computer - 27% disclosed any type of IPV in person - Out of 85 women who disclosed IPV via computer -71% disclosed also in person -Out of 91 women who disclosed with either computer or in person -36% disclosed via the computerized tool but did not disclose in person -7% disclosed IPV in person to the provider but not on the computer - According to patient feedback, the use of both FTF and Computerized should be used together
Choo E. K. et al. (2016) [59]	women aged 18 to 59 reporting both drug use and IPV **Setting**: Emergency Department *N* = 40 women in total	**Study Type**: RCT (2 arms) **Intervention**: Tablet based education modules for IPV (B-SAFER) with content on drug use and IPV + phone booster **Duration**: one session for the web component; 2-weeks for the Booster **Follow-up**: NA **Intervention group**: 21 women **Primary Outcomes and Measurements Tools:** Primary Satisfaction Outcomes 8-item Client Satisfaction Questionnaire; 10-item Systems Usability Scale (SUS)	Control group: 19 B-SAFER (with a content of fire safety) + phone booster	Mean usability score (SUS): 83.5 (95% CI 78.1–88.9) out of a possible 100. Mean overall satisfaction score (CSQ-8) was 27.7 (95% CI 26.3–29.1) out of a possible 32.
Constantino. R. E. et al. (2015) [60]	Women -ages 18 or older -English-speaking -Have basic literacy skills -Not living with perpetrator -Has experienced IPV in past 18 months **Setting**: Neighborhood Legal Services Association; Family Court waiting area; A Women’s Center and Shelter *N* = 32 women	**Intervention Type:** RCT (3 arms) **Intervention**: ONLINE-HELLP modules via email or face-to-face per week **Duration**: 6 weeks (once a week) **Follow-up**: NA **Intervention group**:11 women **Primary Outcomes and Measurement Tools** -IPV Experience Questionnaire (IPVEQ), -Availability of personal support: Personal Resource Questionnaire (PRQ) -Perceived availability of interpersonal and community support: Interpersonal Support Evaluation List (ISEL) -Anxiety, anger and depression: The PROMIS version 1.0 short form	ARM #2 = 6 FTF-HELLP modules in person (face-to-face) Size:10 women ARM #3 = Waitlist/Control group: no intervention Size: 11women	•At baseline, (62%) reported being in physical pain due to IPV •Anxiety, depression, ISEL all showed significant improvements
Eden. K. B. et al. (2015) [61].	women aged 18 years or older English-speaking previous history of IPV **Setting**: women in the general community in 4 states *N* = 708 women	**Intervention Type**: RCT (2 arms) **Intervention**: IRIS Online Interactive safety decision aid with personalized safety plan **Duration:** One use **Follow-up**: NA **Intervention Group**: 354 **Primary Outcome and Measurement Tools:** Decisional conflict: Decisional Conflict Scale (DCS)	Control group: 543 Online Usual safety planning Resource website	• After just one online session: intervention women had significantly lower total decisional conflict than control • no statistically significant difference between control and intervention groups on changes in feeling uninformed
Fincher D. (2015) [62]	Low-income African American Women receiving Special Supplemental Nutrition Program for Women, Infants, and Children (WIC) − 18 years old, -eligible to receive WIC services, -English speaking, -literate **Setting**: Special Supplemental Nutrition Program for Women, Infants, and Children (WIC) *N* = 368	Study Type: RCT 2-arm Intervention: via computer-assisted self interview (CASI) Duration: 2 months (July 17, 2012, and September 21, 2012) Follow-up: 2 weeks (ask about experience with and preference of screening method) Intervention Group: 117 (computed as 48.1% of N) **Primary Outcome and Measurement Tools:** general health behaviors, tobacco use, alcohol use: TWEAK: Tolerance, Worried, Eyeopener, Amnesia, Cut down substance use (Drug Abuse Screening Test IPV victimization: Revised Conflict Tactics Scales–Short Form (CTS2S) 12 dichotomous (yes, no) outcomes for -disclosure of lifetime and prior-year: (a) negotiation skills, (b) exposure to psychological IPV, (c) exposure to physical IPV, (d) exposure to sexual IPV, (e) exposure to any IPV (psychological, physical, or sexual), (f) IPV related injury	Control group: 251 (computed as N-117) face-to-face interview (FTFI).	Women screened via FTFI reported significantly more lifetime and prior year negotiation and more prior year verbal, sexual, and any IPV than CASI-screened women 117 women completed follow-up (3.8% of sample) Face-to-Face more effective for IPV disclosure
Fiorillo. D. et al. (2017) [63].	-women ages 18 years or older -fluent in English -experience of trauma in the form of sexual or physical abuse -have high level of psychological distress (score of 4+ on the binary version of the 12-item General Health Questionnaire) **Setting**: local mental health and community agencies *N* = 25	**Intervention Type**: Open trial, without control and randomization **Intervention**: web-based ACT (acceptance and Commitment Therapy) focused specifically for treatment of PTSD in survivors of interpersonal trauma **Duration**: 6 weeks (6 sessions) **Follow-up**: NA **Intervention Group**: 25 women **Primary Outcomes and Measurement Tools**: exposure to trauma: Life Events Checklist (LEC-5); Distress: General Health Questionnaire (GHQ) Stressful Life Events Screening Questionnaire (SLESQ) PTSD Checklist (PCL-5) Depression, Anxiety and Stress Scale (DASS) **Secondary Outcomes and Measurement Tools:** Knowledge of ACT (ACT Knowledge Quest) psychological flexibility (Acceptance and Action Questionnaire-II (AAQ-II))	Control Group: Same as Intervention Group	• Attrition: 16% (84% completed the treatment and post-treatment assessments) • Significant improvements in targeted outcomes (PTSD, depression, anxiety) upon completion of the 6-session web-based intervention better ACT knowledge and psychological flexibility
Ford-Gilboe M et al. (2020) [64]	Women, 19 years or older who experienced IPV in the previous 6 months. **Setting:** community settings (e.g. libraries) *N* = 531 women	**Study Type**: RCT (2 arms) **Intervention**: tailored, interactive online safety and health intervention **Duration**: 12 months **Follow-up**: 3, 6, 12 months **Intervention group**: *n* = 267 women **Primary Outcomes and Measurements Tools:** Primary: depressive symptoms (CESD-R) and PTSD symptoms (PCL-C) Secondary: helpfulness of safety actions, confidence in safety planning, mastery, social support, experiences of coercive control, and decisional conflict	Control Group: *n* = 264 non-tailored version of the interactive online safety and health intervention	Both groups improved on depression and on all secondary outcomes The tailored intervention had greater positive effects for women (1) with children under 18 living at home; (2)reporting more severe violence; (3)living in medium-sized and large urban centers; (4)and not living with a partner
Gilbert. L. et al. (2016) [65].	women aged 18 years or older Substance-abusers Have at least 1 HIV risk factor Engage in unprotected intercourse **Setting**: multiple community corrections sites *N* = 306 women	**Intervention Type**: RCT (3 arms) **Intervention**: 4 group sessions with computerized WORTH, self-paced IPV prevention modules **Duration**: 1 week **Follow-up**: 6 months, and 12-months **Intervention group**:103 women **Primary Outcome and Measurements Tools:** the risk of different types of IPV victimization: 8-item version of the Revised Conflict Tactics Scale **Secondary Outcome and Measurements Tools** Illicit drugs ever and within the past 90 days.: Risk Behavior Assessment	ARM 2: 4 weekly traditional group sessions covering same material without computersSize:101 womenControl group/ARM 3: 4 weekly sessions for wellness promotion Size: 102 women	-Computerized WORTH participants were 62% less likely to report experiencing any physical IPV at the 12-month follow-up; 76% less likely to report injurious IPV; 78% less likely to report severe sexual IPV No difference was observed between computerized WORTH and traditional WORTH
Glass. N., Eden.K. et al. (2010) [66]	Participants Female Patients who Spoke English or Spanish 18 years of age or older reported physical and/or sexual violence within a relationship in the previous year **Setting**: domestic violence shelters or domestic violence support groups *N* = 90 women	**Intervention Type:** Open trial, without control and randomization **Intervention**: Computerized safety decision aid **Duration**: NA **Follow-up**: NA **Intervention Group**: 90 (Age 17 to 63) **Primary Outcomes and Measurement Tools** The Decisional Conflict Scale (DCS) Feeling Supported Certainty about safety plans Knowledge of options Clear Priorities **Other tools** -Danger Assessment (DA)	Control group: Same as Intervention Group Control Size:90 participants	-Mean DA at baseline was (18.14), meaning extreme danger during the last year -Post intervention statistically significant measures - participants felt more supported in their decision - reported less total decisional conflict - No significant difference - Certainty about their safety plans - Knowledge of their options - Clear Values/priorities − 60% reported having made a safety plan − 76% included a plan to leave the relationship **limitations:** - participants were already in a help seeking phase (shelter, support groups) - More than 90% of these participants reported they had left the abusive relationship in the past year
Hassija C. and Gary MJ (2011) [67]	Age 19–52 referred to from a distal domestic violence and rape crisis centers **Setting**: Trauma Telehealth Treatment Clinic *N* = 15	Study Type: Open trial, without control and randomization Intervention: Treatment via videoconferencing Duration: mostly are one-time consult Follow-up: NA **Primary Outcomes and Measurement Tools** -PTSD severity: Post-traumatic Stress Disorder Checklist (PCL) - DSM IV -Depression symptom severity: The Center for Epidemiological Studies Depression Scale (CES-D), -Client satisfaction: Wyoming Telehealth Trauma Clinic Client Satisfaction Scale (WTICCSS)	Control Group: Same as Intervention Group	Large reductions on measures of PTSD and depression symptom severity High degree of satisfaction
Hegarty K et al. (2019) [68]	Women, 16–50 years who had screened positive for any form of IPV or fear of a partner in the 6 months before recruitment. **Setting:** community settings *N* = 422 women	**Study Type**: RCT (2 arms) **Intervention**: I-DECIDE: Website on healthy relationships, abuse and safety, and relationship priority setting, and a tailored action plan. **Duration**: 3–60 min **Follow-up**: 6 months, 12 months**Intervention group**: *n* = 227 women **Primary Outcomes and Measurements Tools:** - Self-efficacy (Generalized Self-Efficacy Scale) - depression (Center for Epidemiologic Studies Depression Scale—Revised)	Control Group: *n* = 195 **Static intimate partner violence information** (5 min duration)	Women in the control group had higher self-efficacy scores at 6 months and 12 months than did women in the intervention group No between group differences in depression at 6 months or 12 months Qualitative: Qualitative findings indicated that participants found the intervention supportive and a motivation for action.
Humphreys. J. et al. (2011) [69]	Pregnant women who presented for routine prenatal care who also reported being at risk for intimate partner violence (IPV) English-speaking 18 years or older Fewer than 26 weeks pregnant Receiving prenatal care at one of the participating clinics, Not presenting for their first prenatal visit **Setting**: prenatal clinics Urban *N* = 50	**Intervention Type**: RCT (2 arms) **Intervention**: Video Doctor that generates: Provider Cueing + patient education sheet **Duration**: NA **Follow-up**: 1 month during next monthly routine visits **Intervention Group**: 25 **Outcomes and Measurements Tools** - IPV: Abuse Assessment Screen -occurrence of patient–provider discussion of IPV risk: Abuse Assessment Screen-participants’ perceived helpfulness of the discussion. -intention to make changes: seriously thinking of making a change within next 30 days or 6 months	Control group: *N* = 25 usual prenatal care	Video Doctor plus Provider Cueing significantly increases health care provider–patient IPV discussion -At baseline: 81.8% of Intervention group participants reported IPV vs. 16.7% control group (significant) -At 1-month follow-up: 70.0% of Intervention group participants reported IPV vs. 23.5% control group (significant) - 90% of intervention participants were significantly more likely to have IPV risk discussion with their providers at one or both visits compared 23.6% of control group participants who received usual care - 32 participants reported the intention to make changes regarding IPV within the 30 days to 6 Months vs. 14 participants in control
Koziol-McLain. J. et al. (2018) [9].	women experience IPV in the last 6 months; aged 16 years or older; have access to safe: computer, email address, and internet **Setting**: online Ads (info from previous publication) *N* = 412 women total Note: 27% Maori(indigenous)	**Intervention Type:** RCT (2 arms) **Intervention**: Web-based decision aid (i-safe -individualized website) who experienced IPV during the last 6 months **Duration**: 12 months (September 2012 to September 2014) **Follow-up:** 3,6, and 12 months **Intervention Group** = 202 **Primary Outcomes and Measurements Tools:** CESD-R: self-reported mental health (depression) SVAWS: Severity of Violence Against Women Scale	standardized, non-individualized web-based information Control Group = 210 women	-Attrition: 35% -individualized Web-based isafe decision aid -Intervention group had 12% increase in safety behaviors, control group had 9% increase − 78% stated isafe provided them with new skills − 91% stated isafe provided them with useful information -No significant differences in SVAWs score nor CESD-R score overall -The interactive, individualized Web-based isafe decision aid was effective in reducing IPV exposure limited to indigenous Māori women. -reduction of depression was significant for Maori women post trial; but was not observed at 3 and 6 months
MacMillan. H.L. et al. (2006) [70].	Women ages 18 to 64 years English-speaking **Setting**: 2 Emergency Departments, 2 Family practices, 2 Women’s health clinics *N* = 2416 women	**Intervention Type:** Cluster RCT (3 arms) **Intervention**: Screening: Face-to-Face, Computer based, Paper based **Duration**: 8 months (May 2004 to January 2005) **Follow-up**: NA **Intervention Group:** Computer Based Screening (769 participants) **Primary Outcomes and Measurements Tools:** -Prevalence of IPV (3 scales used): -Partner Violence Screen (PVS), -Woman Abuse Screening Tool (WAST) -Composite Abuse Scale (CAS) -Extent of missing data -Participant preference	Control group(s): (1) Face-to-face interview with a health care provider (853 participants) (2) written self-completed questionnaire (839 participants)	−12-month prevalence of IPV ranged from 4.1 to 17.7%, depending on screening method, instrument, and health care setting -No statistically significant main effects on prevalence were found for method or screening instrument, - A significant interaction between method and instrument was found -Face-to-face approach was least preferred by participants
McNutt L. A.et al. (2005) [71]	Women, 18 to 44 years **Setting:** community health center *N* = 211 women	**Study Type**: RCT (3 arms) **Intervention**: Short Computer screening **Duration**: one session for the web component; 2-weeks for the Booster **Follow-up**: NA **Intervention group**: n: unknown **Primary Outcomes and Measurements Tools:** Sensitivity analysis	Control Group: n: unknown Arm2: Short Face-to-face screening with a nurse Arm3: Long computer screening	The two computerized screening protocols were more sensitive and less or similarly specific than documented nursing staff screening
Renker, P. R., & Tonkin, P. (2007) [72]	Postpartum Women at Level III maternity units in two hospitals *N* = 519	Study Type: Cross-sectional Survey Intervention: Computerized Questionnaire + voice and Video Duration: N/A Follow-up: NA **Primary Outcomes and Measurements Tools:** (1) Participants’ evaluations of the A-CASI interview to screen for perinatal abuse(2) Participants’ preferences for mode of violence screening (face-to-face, written form, or computer) (3)Participants’ perceptions of the truthfulness and completeness of their answers on the A-CASI (4) Anonymity associated with the A-CASI affect women’s perceptions of their truthfulness when responding to the questions? (5) the relationship between the women’s abuse status and preferences for mode of screening, self-report of truthfulness, and evaluation of the A-CASI interview (6) The relationship of age, source of healthcare, and race to preference for mode of screening, self-report of truthfulness, and evaluation of the A-CASI interview	No Control Group	Women overwhelmingly preferred computerized screening for violence over face-to-face and written formats. Including computer violence screening for all women, regardless of point of care, age, economic, or racial and ethnic background.
Rhodes et al. (2002) [74]	Women and Men 18–65 Presented for emergency care with a nonurgent complaint Triaged into the lowest 2 categories of our 5-level triage system **Setting**: Urban emergency department *N* = 248 (170 women, 78 men)	**Study Type**: RCT (2 arms) **Intervention**: Computer screening (generate health advice and patient risk summaries physicians) **Duration**: NA **Follow-up**: NA **Intervention Group** 248 (women and men) (170 women) **Primary Outcomes and Measurements Tools:** -Abuse Assessment Screen (AAS) -Partner Violence Screen (PVS) -items from Improving Health Care Response to Domestic Violence: A Resource Manual for Health Care Providers	Control Group: 222 (women and men) usual care	**Disclosure** Disclosure in the Intervention Group was significantly higher than Control: 19 cases (17 women + 2 men) out of potential 83 potential cases vs. 1 case in control (no gender reported) **Detection** Substantially higher detection rate of IPV in intervention group compared to control group; **but it did not guarantee charting and follow-up by the treating physician**
Rhodes et al. (2006) [73]	women ages 18 to 65 years non-emergent female patients **Setting**: Emergency Departments (Urban and Suburban) *N* = 1281 women	**Study Type:** RCT (2 arms) **Intervention**: self-administered computer-based health risk assessment, with a prompt for the health care provider Duration: 7 months (June 2001 and December 2002) Follow-up: NA **Intervention Group**: 637 women **Primary Outcomes and Measurements Tools:** (assessed by audiotape analysis) Abuse Assessment Screen (AAS) Partner Violence Screen (PVS) -rates of discussion of DV, -patient disclosure of DV to the health care provider, -evidence of DV services provided during the visit (safety assessment, counseling by the health care provider or social worker, or referrals to DV resources) **Secondary Outcomes** -Medical chart documentation of DV screening (positive or negative) -DV “case finding” (chart documentation of current or past DV), -overall patient satisfaction	Control group: 644 usual care	-Rates of current DV risk on exit questionnaire were 26% in the urban ED and 21% in the suburban ED Primary Outcomes - In the urban ED, the computer prompt increased rates of DV discussion, disclosure, and services provided. - Women at the suburban site and those with private insurance or higher education were much less likely to be asked about experiences with abuse. - Only 48% of encounters with a health care provider prompt regarding potential DV risk led to discussions. - Inquiries about, and disclosures of, abuse were associated with higher patient satisfaction with care.
Scribano et al. (2011) [75]	Caregivers (male and female) of children in a pediatric ED **Setting:** Pediatric Emergency Departments *N* = 13,057 computerized screens	**Study Type**: Observational **Intervention**: Home safety screening kiosks **Duration**: 15 months (October 1, 2008, to December 31, 2009) **Follow-up**: NA **Intervention Group:** 13,057 computerized screens in an ED **Primary Outcomes and Measurements Tools:** Partner Violence Screen (1) evaluate the feasibility of adjunctive, caregiver-initiated computer technology in a pediatric ED visit to determine home safety risks (2) determine the system reliability (technology failure rate).	Control group: Face-to-Face screening	13.7% among those who used the kiosks were positive for IPV High adoption of the e-screening kiosk High Reliability of Technology (downtime 4.2% of days) Need of champions to increase adoption rate
Sprecher. A. G. et al. (2004) [76].	All female patients from the 1996 ED database **Setting**: A Medical Center Visits N = 19,830 patient’s data	**Type of Study**: Observational (retrospective) **Intervention**: Neural Network Model (The model was a two-layer network without any hidden processing layers. Both the input and output layers consisted of 100 elements yielding 10,000 connections between the elements.) **Duration**: NA **Follow-up**: NA **Intervention Group:** 19,830 records **Primary Outcomes and Measurements Tools:** Ability of a neural network model to identify potential victims of IPV using patient’s data	No control group	- The Neural Network identified 231 of 297 known IPV victims (sensitivity 78%) - The Neural Network categorized 2234 false-positive patients out of 19,533 IPV-negative patients (specificity 89%)
Thomas. C.R. et al. (2005) [77].	women referred by mental health screening and treatment of domestic violence **Setting**: rural women’s shelter program *N* = 35 women in total	**Intervention Type**: Open trial, without control and randomization **Intervention:** Psychiatric evaluation and treatment provided using telepsychiatry **Duration:** NA **Follow-up:** NA **Intervention group**: 38 women **Primary Outcomes and Measurements Tools:** Descriptive Patient satisfaction questionnaire Improving mental health services for victims of domestic violence	No control group	•most commonly identified disorders were anxiety and major affective disorders, followed by substance use disorders Goal reached: Out of the 38 cases screened, 35 (92%) completed the evaluation, 31 (82%) began treatment, and 20 (53%) were transferred to ongoing outpatient care.
Trautman. D. E. et al. (2007) [78].	women ages 18 years or older **Setting**: Emergency Department *N* = 1005 women in total	**Study Type**: RCT (2 arms) **Intervention**: Computer-based health survey for IPV screening **Duration**: 6 weeks **Follow-up**: NA **Intervention group**: 411 women **Primary Outcomes and Measurements Tools:** **Outcomes** screening, detection, referral and service rates	Control group: 594 usual intimate partner violence care (screened voluntarily by ED providers and documented in medical record).	- 99.8% of intervention participants were screened for intimate partner violence compared to 33% of control participants -computer-based health survey detected 19% intimate partner violence positive whereas usual care detected 1% -Subjects in the intervention group received intimate partner violence services more than subjects in the usual care (4% vs 1%)

^a^*Legend*: 1 = Random sequence used; 2 = Allocation concealed; 3 = Study participants blinded; 4 = Research personnel blinded; 5 = Outcome assessment blinded; 6 = Attrition low; 7 = Non-selective reporting

**Table 5:** Primary outcomes in the included studies

Study	Screening and Disclosure					IPV Prevention							ICT Suitability		Support				Mental Health								
	Count	NVQ	IPVEQ	DA/DA-R	PVS	AI	AAS	WAST	CAS	CTS2	CTS2S	SVAWS	CSQ-8	SUS	PRQ	ISEL	DCS	GSE	PROMIS	LEC-5	SLESQ	DASS	PCL	CESD-R	PCL-C	CESD	SCL-90-R
Ahmad et al.	**✓**																										
Bacchus et al.	**✓**																										
Braithwaite et a.										**✓**																	
Chang et al.		**✓**																									
Choo et al.													**✓**	**✓**													
Constantino et al.			**✓**												**✓**	**✓**			**✓**								
Eden et al.				**✓**													**✓**										
Fincher et al.										**✓**																	
Fiorillo et al.																				**✓**	**✓**	**✓**	**✓**				
Ford-Gilboe et al. (2020)																								**✓**	**✓**		
Gilbert et al.										**✓**																	
Glass et al.																	**✓**										
Hassija et al.																							**✓**			**✓**	
Hegarty et al. (2019)																		**✓**						**✓**			
Humphreys et al.	**✓**						**✓**																				
Koziol-McLain et al.												**✓**												**✓**			
MacMillan					**✓**			**✓**	**✓**																		
McNutt et al.	**✓**																										
Renker & Tonkin		**✓**																									
Rhodes et al. (2002)					**✓**		**✓**																				
Rhodes et al. (2006)					**✓**		**✓**																				
Scribano et al.	**✓**																										
Sprecher et al.						**✓**																					
Thomas et al.																											**✓**
Trautman et al.	**✓**																										

*Legend*: *CTS2* Revised Conflict Tactics Scale, *CTS2S* Revised Conflict Tactics Scales–Short Form, *CSQ-8* Client Satisfaction Questionnaire, *SUS* Systems Usability Scale, *IPVEQ* IPV Experience Questionnaire, *PRQ* the Personal Resource Questionnaire, *ISEL* the Interpersonal Support Evaluation List, *PROMIS* Patient-Reported Outcomes Measurement Information System, *DA* Danger Assessment, *DA-R* DA-Revised, *DCS* Decisional Conflict Scale, *LEC-5* Life Events Checklist, *SLESQ* Stressful Life Events Screening Questionnaire, *PCL-5* PTSD Checklist, *DASS* Depression, Anxiety and Stress Scale, *CESD-R* Center for Epidemiologic Studies Depression Scale Revised, *PCL-C* PTSD checklist, Civilian Version, *GSE* Generalized Self-Efficacy Scale, *SVAWS* Severity of Violence Against Women Scale, *PVS* Partner Violence Screen, *WAST* Woman Abuse Screening Tool, *CAS* Composite Abuse Scale, *AAS* Abuse Assessment Screen, *SCL-90-R* Symptom Checklist-90-R, *NVQ* Non-Validated Questionnaire, *AI* Artificial Intelligence

## Abbreviations

IPV: Intimate Partner Violence
ICT: Information and Communication Technologies

## References

[1] The Centers for Disease Control and Prevention (CDC) (2018). Intimate partner violence Philadelphia.

[2] UN News Center (2014). UN sounds alarm to end ‘global pandemic’ of violence against women.

[3] Human Rights Council. Work of the human rights council (2006 – present) and the commission on human rights (until 2006). New York: United Nations; 2006. [updated March 15th, 2006. Available from: http://www.un.org/womenwatch/daw/vaw/v-hrc.htm.

[4] Garcia-Moreno C, Watts C (2011). Violence against women: an urgent public health priority. Bull World Health Organ.

[5] Nasir K, Hyder AA (2003). Violence against pregnant women in developing countries: review of evidence. Eur J Pub Health.

[6] Heise LL, Raikes A, Watts CH, Zwi AB (1994). Violence against women: a neglected public health issue in less developed countries. Soc Sci Med.

[7] Campbell J, García-Moreno C, Sharps P (2004). Abuse during pregnancy in industrialized and developing countries. Violence Against Women.

[8] The Roeher Institute (2004). Violence against women with disabilities.

[9] Koziol-McLain J, Vandal AC, Wilson D, Nada-Raja S, Dobbs T, McLean C (2018). Efficacy of a web-based safety decision aid for women experiencing intimate partner violence: randomized controlled trial. J Med Internet Res.

[10] Poushter J (2016). Smartphone ownership and internet usage continues to climb in emerging economies.

[11] Neil AL, Batterham P, Christensen H, Bennett K, Griffiths KM (2009). Predictors of adherence by adolescents to a cognitive behavior therapy website in school and community-based settings. J Med Internet Res.

[12] Usher W (2009). General practitioners' understanding pertaining to reliability, interactive and usability components associated with health websites. Behav Inform Technol.

[13] Vázquez G, Roca J, Blanch L (2009). The challenge of web 2.0-based. Med Intensiva.

[14] Zarinah MK, Siti SS (2009). A web-based requirements elicitation tool using focus group discussion in supporting computer-supported collaborative learning requirements development. Int J Comput Internet Manag.

[15] Abbott R (2010). Delivering quality-evaluated healthcare information in the era of web 2.0: design implications for Intute: health and life sciences. Health Inf J.

[16] Kuosmanen L, Jakobsson T, Hyttinen J, Koivunen M, Välimäki M (2010). Usability evaluation of a web based patient information system for individuals with severe mental health problems. J Adv Nurs.

[17] Wanner M, Martin-Diener E, Bauer G, Braun-Fahrlander C, Martin BW (2010). Comparison of trial participants and open access users of a web-based physical activity intervention regarding adherence, attrition, and repeated participation. J Med Internet Res.

[18] Gluck TM, Maercker A (2011). A randomized controlled pilot study of a brief web-based mindfulness training. BMC Psychiatry.

[19] Maret P, Vercouter L, El Morr C (2011). Special issue on web intelligence and virtual communities. Editorial. Int J Netw Virtual Organ.

[20] Nijhof SL, Bleijenberg G, Uiterwaal C, Kimpen JLL, van de Putte EM (2011). Fatigue in teenagers on the interNET - the FITNET trial. a randomized clinical trial of web-based cognitive behavioural therapy for adolescents with chronic fatigue syndrome: study protocol. ISRCTN59878666. BMC Neurol.

[21] Storch EA, Caporino NE, Morgan JR, Lewin AB, Rojas A, Brauer L (2011). Preliminary investigation of web-camera delivered cognitive-behavioral therapy for youth with obsessive-compulsive disorder. Psychiatry Res.

[22] Krusche A, Cyhlarova E, King S, Williams JM. Mindfulness online: a preliminary evaluation of the feasibility of a web-based mindfulness course and the impact on stress. BMJ Open. 2012;2(3):e000803. 10.1136/bmjopen-2011-000803 PMC3358627 22614170

[23] Radhu N, Daskalakis ZJ, Arpin-Cribbie CA, Irvine J, Ritvo P (2012). Evaluating a web-based cognitive-behavioral therapy for maladaptive perfectionism in university students. J Am Coll Heal.

[24] Barnett S, Jones SC, Bennett S, Iverson D, Bonney A (2013). Usefulness of a virtual community of practice and web 2.0 tools for general practice training: experiences and expectations of general practitioner registrars and supervisors. Aust J Prim Health.

[25] Fletcher P, Poon A, Pearce B, Comber P. Practical web traffic analysis: standards, privacy, techniques, and results. New York: Apress; 2013.

[26] Hsu SH, Chang JW, Lee CC (2013). Designing attractive gamification features for collaborative storytelling websites. Cyberpsychol Behav Soc Netw.

[27] Krusche A, Cyhlarova E, Williams JM (2013). Mindfulness online: an evaluation of the feasibility of a web-based mindfulness course for stress, anxiety and depression. BMJ Open.

[28] Li TM, Chau M, Wong PW, Lai ES, Yip PS (2013). Evaluation of a web-based social network electronic game in enhancing mental health literacy for young people. J Med Internet Res.

[29] Allam A, Kostova Z, Nakamoto K, Schulz PJ (2015). The effect of social support features and gamification on a web-based intervention for rheumatoid arthritis patients: randomized controlled trial. J Med Internet Res.

[30] Buckingham CD, Adams A, Vail L, Kumar A, Ahmed A, Whelan A (2015). Integrating service user and practitioner expertise within a web-based system for collaborative mental-health risk and safety management. Patient Educ Couns.

[31] Davis JM, Manley AR, Goldberg SB, Stankevitz KA, Smith SS (2015). Mindfulness training for smokers via web-based video instruction with phone support: a prospective observational study. BMC Complement Alternat Med.

[32] Guille C, Zhao Z, Krystal J, Nichols B, Brady K, Sen S (2015). Web-based cognitive behavioral therapy intervention for the prevention of suicidal ideation in medical interns a randomized clinical trial. Jama Psychiatry.

[33] Heck NC, Saunders BE, Smith DW (2015). Web-based training for an evidence-supported treatment: training completion and knowledge Acquisition in a Global Sample of learners. Child Maltreat.

[34] Khanna MS, Kendall PC (2015). Bringing technology to training: web-based therapist training to promote the development of competent cognitive-behavioral therapists. Cogn Behav Pract.

[35] Lappalainen P, Langrial S, Oinas-Kukkonen H, Tolvanen A, Lappalainen R (2015). Web-based acceptance and commitment therapy for depressive symptoms with minimal support: a randomized controlled trial. Behav Modif.

[36] Muessig KE, Nekkanti M, Bauermeister J, Bull S, Hightow-Weidman LB (2015). A systematic review of recent smartphone, internet and web 2.0 interventions to address the HIV continuum of care. Curr HIV/AIDS Rep.

[37] Levin ME, Hayes SC, Pistorello J, Seeley JR (2016). Web-based self-help for preventing mental health problems in universities: comparing acceptance and commitment training to mental health education. J Clin Psychol.

[38] Bloss CS, Wineinger NE, Peters M, Boeldt DL, Ariniello L, Kim JY (2016). A prospective randomized trial examining health care utilization in individuals using multiple smartphone-enabled biosensors. PeerJ..

[39] Luxton DD, McCann RA, Bush NE, Mishkind MC, Reger GM (2011). mHealth for mental health: integrating smartphone technology in behavioral healthcare. Prof Psychol Res Pract.

[40] Sundaram P, Wolfersberger J, Jenkins M. Acting on the Evolution of the Canadian Smartphone User March 2014: catalyst.ca; 2014 [Available from: http://catalyst.ca/wp-content/uploads/Catalyst_Canadian-Smartphone.pdf.

[41] Torous J, Chan SR, Yee-Marie Tan S, Behrens J, Mathew I, Conrad EJ (2014). Patient smartphone ownership and interest in Mobile apps to monitor symptoms of mental health conditions: a survey in four geographically distinct psychiatric clinics. JMIR Mental Health.

[42] Torous J, Friedman R, Keshavan M (2014). Smartphone ownership and interest in Mobile applications to monitor symptoms of mental health conditions. JMIR mHealth uHealth.

[43] Dieterle B (2015). Designing smartphone apps for at risk populations: domestic violence survivors and user experience. Proceedings of the 33rd annual international conference on the Design of Communication.

[44] El Morr C, Saleh S, Ammar W, Natafgi N, Kazandjian K (2014). A health VC for chronic disease management in a global context. Third international conference on Global Health challenges, Global Health 2014, august 24–28, 2014.

[45] El Morr C. Mobile virtual communities in healthcare: the chronic disease management case. In: Mohammed S, Fiadi J, editors. Ubiquitous health and medical informatics: the ubiquity 20 trend and beyond: Hershey:IGI Global; 2010. p. 258–74.

[46] Ahmad F, El Morr C, Ritvo P, Othman N, Moineddin R, MVC Team (2020). An eight-week, web-based mindfulness virtual community intervention for Students' mental health: randomized controlled trial. JMIR Ment Health.

[47] El Morr C, Maule C, Ashfaq I, Ritvo P, Ahmad F. Design of a Mindfulness Virtual Community: a focus-group analysis. Health Inf J. 2019;1460458219884840:e2. 10.1177/1460458219884840 31709878

[48] El Morr C, Ginsburg L, Nam S, Woollard S (2017). Assessing the performance of a modified LACE index (LACE-rt) to predict unplanned readmission after discharge in a community teaching hospital. Interact J Med Res.

[49] Mancini F (2013). New technology and the prevention of violence and conflict. Int J Secur Dev.

[50] Mahajan M, Reddy K, Rajput M (2016). Design and implementation of a rescue system for safety of women. 2016 international conference on wireless communications, signal processing and networking (WiSPNET).

[51] Bahia K, Suardi S (2019). The state of Mobile internet connectivity 2019.

[52] Marcolino MS, Oliveira JAQ, D'Agostino M, Ribeiro AL, Alkmim MBM, Novillo-Ortiz D (2018). The impact of mHealth interventions: systematic review of systematic reviews. JMIR Mhealth Uhealth.

[53] Eisenhut K, Sauerborn E, Garcia-Moreno C, Wild V (2020). Mobile applications addressing violence against women: a systematic review. BMJ Glob Health.

[54] Morrison A, Polisena J, Husereau D, Moulton K, Clark M, Fiander M (2012). The effect of english-language restriction on systematic review-based meta-analyses: a systematic review of empirical studies. Int J Technol Assess Health Care.

[55] Ahmad F, Hogg-Johnson S, Stewart DE, Skinner HA, Glazier RH, Levinson W (2009). Computer-assisted screening for intimate partner violence and control a randomized trial. Ann Intern Med.

[56] Bacchus LJ, Bullock L, Sharps P, Burnett C, Schminkey DL, Buller AM (2016). Infusing technology into perinatal home visitation in the United States for women experiencing intimate partner violence: exploring the interpretive flexibility of an mHealth intervention. J Med Internet Res.

[57] Braithwaite SR, Fincham FD (2014). Computer-based prevention of intimate partner violence in marriage. Behav Res Ther.

[58] Chang JC, Dado D, Schussler S, Hawker L, Holland CL, Burke JG (2012). In person versus computer screening for intimate partner violence among pregnant patients. Patient Educ Couns.

[59] Choo EK, Zlotnick C, Strong DR, Squires DD, Tape C, Mello MJ (2016). BSAFER: a web-based intervention for drug use and intimate partner violence demonstrates feasibility and acceptability among women in the emergency department. Subst Abus.

[60] Constantino RE, Braxter B, Ren D, Burroughs JD, Doswell WM, Wu L (2015). Comparing online with face-to-face HELPP intervention in women experiencing intimate partner violence. Issues Ment Health Nurs.

[61] Eden KB, Perrin NA, Hanson GC, Messing JT, Bloom TL, Campbell JC (2015). Use of online safety decision aid by abused women: effect on decisional conflict in a randomized controlled trial. Am J Prev Med.

[62] Fincher D, VanderEnde K, Colbert K, Houry D, Smith LS, Yount KM (2015). Effect of face-to-face interview versus computer-assisted self-interview on disclosure of intimate partner violence among African American women in WIC clinics. J Interpers Violence.

[63] Fiorillo D, McLean C, Pistorello J, Hayes SC, Follette VM (2017). Evaluation of a web-based acceptance and commitment therapy program for women with trauma-related problems: a pilot study. J Contextual Behav Sci.

[64] Ford-Gilboe M, Varcoe C, Scott-Storey K, Perrin N, Wuest J, Wathen CN (2020). Longitudinal impacts of an online safety and health intervention for women experiencing intimate partner violence: randomized controlled trial. BMC Public Health.

[65] Gilbert L, Goddard-Eckrich D, Hunt T, Ma X, Chang M, Rowe J (2016). Efficacy of a computerized intervention on HIV and intimate partner violence among substance-using women in community corrections: a randomized controlled trial. Am J Public Health.

[66] Glass N, Eden KB, Bloom T, Perrin N (2010). Computerized aid improves safety decision process for survivors of intimate partner violence. J Interpers Violence..

[67] Hassija C, Gray MJ (2011). The effectiveness and feasibility of videoconferencing technology to provide evidence-based treatment to rural domestic violence and sexual assault populations. Telemed E-Health.

[68] Hegarty K, Tarzia L, Valpied J, Murray E, Humphreys C, Taft A (2019). An online healthy relationship tool and safety decision aid for women experiencing intimate partner violence (I-DECIDE): a randomised controlled trial. Lancet Public Health.

[69] Humphreys J, Tsoh JY, Kohn MA, Gerbert B (2011). Increasing discussions of intimate partner violence in prenatal care using video doctor plus provider cueing: a randomized, controlled trial. Womens Health Issues.

[70] MacMillan HL (2006). Approaches to screening for intimate partner violence in health care settings. JAMA..

[71] McNutt LA, McCauley J, Campbell J, Abushomar H, Ford D (2005). Validity of touch screen computers for preventive services screening in primary care: assessment of screening for intimate partner violence. Am J Epidemiol.

[72] Renker PR, Tonkin P (2007). Postpartum women's evaluations of an audio/video computer-assisted perinatal violence screen. Cin-Comput Inf Nurs.

[73] Rhodes KV, Drum M, Anliker E, Frankel RM, Howes DS, Levinson W (2006). Lowering the threshold for discussions of domestic violence: a randomized controlled trial of computer screening. Arch Intern Med.

[74] Rhodes KV, Lauderdale DS, He T, Howes DS, Levinson W (2002). “Between me and the computer”: increased detection of intimate partner violence using a computer questionnaire. Ann Emerg Med.

[75] Scribano PV, Stevens J, Marshall J, Gleason E, Kelleher KJ (2011). Feasibility of computerized screening for intimate partner violence in a pediatric emergency department. Pediatr Emerg Care.

[76] Sprecher AG, Muelleman RL, Wadman MC (2004). A neural network model analysis to identify victims of intimate partner violence. Am J Emerg Med.

[77] Thomas CR, Miller G, Hartshorn JC, Speck NC, Walker G (2005). Telepsychiatry program for rural victims of domestic violence. Telemed e-Health.

[78] Trautman DE, McCarthy ML, Miller N, Campbell JC, Kelen GD (2007). Intimate partner violence and emergency department screening: computerized screening versus usual care. Ann Emerg Med.

[79] Park SH (2018). Diagnostic case-control versus diagnostic cohort studies for clinical validation of artificial intelligence algorithm performance. Radiology..

[80] El Morr C, Layal M (2019). ICT-based interventions for women experiencing intimate partner violence: research needs in usability and mental health. Stud Health Technol Inform.

[81] Department of Justice. JustFacts: victimization of indigenous women and girls. Ottawa: Government of Canada; 2017. [updated December 15, 2018. Available from: https://www.justice.gc.ca/eng/rp-pr/jr/jf-pf/2017/july05.html.

[82] Klingspohn DM (2018). The importance of culture in addressing domestic violence for first Nation's women. Front Psychol.

[83] Frye V, Hosein V, Waltermaurer E, Blaney S, Wilt S (2005). Femicide in new York City: 1990 to 1999. Homicide Stud.

[84] Oetzel J, Duran B (2004). Intimate partner violence in American Indian and/or Alaska native communities: a social ecological framework of determinants and interventions. Am Indian Alsk Native Ment Health Res.

[85] Sabri B, Njie-Carr VPS, Messing JT, Glass N, Brockie T, Hanson G (2019). The weWomen and ourCircle randomized controlled trial protocol: a web-based intervention for immigrant, refugee and indigenous women with intimate partner violence experiences. Contemp Clin Trials.

[86] Chesser A, Burke A, Reyes J, Rohrberg T (2016). Navigating the digital divide: a systematic review of eHealth literacy in underserved populations in the United States. Inform Health Soc Care.

[87] Philbin MM, Parish C, Pereyra M, Feaster DJ, Cohen M, Wingood G (2019). Health disparities and the digital divide: the relationship between communication inequalities and quality of life among women in a Nationwide prospective cohort study in the United States. J Health Commun.

[88] Mackert M, Mabry-Flynn A, Champlin S, Donovan EE, Pounders K (2016). Health literacy and health information technology adoption: the potential for a new digital divide. J Med Internet Res.

[89] Adler-Milstein J, Holmgren AJ, Kralovec P, Worzala C, Searcy T, Patel V (2017). Electronic health record adoption in US hospitals: the emergence of a digital “advanced use” divide. J Am Med Inform Assoc.

[90] El Morr C, Eftychiou L, Menvielle L, Audrain-Pontevia A-F, Menvielle W (2017). Evaluation frameworks for health virtual communities. The digitization of healthcare.

[91] El Morr C, Subercaze J, Cruz-Cunha MM, Tavares AJ, Simoes R (2010). Knowledge Management in Healthcare. Handbook of research on developments in E-health and telemedicine.

[92] El Morr C. Health care virtual communities. In: Cruz-Cunha MM, Tavares AJ, Simoes R, editors. Handbook of research on developments in E-health and telemedicine. Hershey: IGI Global; 2010. p. 278–98.

[93] Ahmad F, Wang JJ, El Morr C (2018). Online mindfulness interventions: a systematic review. Novel applications of virtual communities in healthcare settings.

[94] El Morr C (2019). Virtual communities, machine learning and IoT: opportunities and challenges in mental Health Research. Int J Extreme Automation Connectivity Healthc.

[95] Goodman LA, Thomas KA, Nnawulezi N, Lippy C, Serrata JV, Ghanbarpour S (2018). Bringing community based participatory research to domestic violence scholarship: an online toolkit. J Fam Violence.

[96] van Gelder NE, van Rosmalen-Nooijens K, Ligthart SA, Prins JB, Oertelt-Prigione S, Lagro-Janssen ALM. SAFE: an eHealth intervention for women experiencing intimate partner violence - study protocol for a randomized controlled trial, process evaluation and open feasibility study. BMC Public Health. 2020;20(1):640. 10.1186/s12889-020-08743-0 PMC7204286 32380972

[97] Crowe A, Overstreet NM, Murray CE. The intimate partner violence stigma scale: initial development and validation. J Interpers Violence. 2019;886260519834095. 10.1177/0886260519834095. 10.1177/0886260519834095 30866696

[98] McCleary-Sills J, Namy S, Nyoni J, Rweyemamu D, Salvatory A, Steven E (2016). Stigma, shame and women's limited agency in help-seeking for intimate partner violence. Glob Public Health.

[99] El Morr C (2018). Introduction to health informatics: a Canadian perspective.

[100] Saleh S, Alameddine M, Farah A, El Arnaout N, Dimassi H, Muntaner C (2018). eHealth as a facilitator of equitable access to primary healthcare: the case of caring for non-communicable diseases in rural and refugee settings in Lebanon. Int J Public Health.

[101] Saleh S, Farah A, Dimassi H, El Arnaout N, Constantin J, Osman M (2018). Using Mobile health to enhance outcomes of noncommunicable diseases Care in Rural Settings and Refugee Camps: randomized controlled trial. JMIR Mhealth Uhealth..

[102] Saleh S, Farah A, El Arnaout N, Dimassi H, El Morr C, Muntaner C (2018). mHealth use for non-communicable diseases care in primary health: patients’ perspective from rural settings and refugee camps. J Public Health (Oxf).

[103] Dimond JP, Fiesler C, Bruckman AS (2011). Domestic violence and information communication technologies. Interact Comput.

[104] Park HA (2016). Health informatics in developing countries: a review of unintended consequences of IT implementations, as they affect patient safety and recommendations on how to address them. Yearb Med Inform.

[105] Ash JS, Berg M, Coiera E (2004). Some unintended consequences of information technology in health care: the nature of patient care information system-related errors. J Am Med Inf Assoc.

[106] Royal Tropical Institute. Technical brief: mHealth for maternal and newborn health in resource-poor and health system settings Sierra Leone. Freetown: Government of Sierra Leone; 2011. [Available from: https://assets.publishing.service.gov.uk/media/57a08adaed915d3cfd000998/technicalbrief-mhealth-SierraLeone.pdf.

[107] Adibi S. mHealth multidisciplinary verticals. Boca Raton: Taylor & Francis; 2014.

[108] Rotheram-Borus MJ, Tomlinson M, Swendeman D, Lee A, Jones E (2012). Standardized functions for smartphone applications: examples from maternal and child health. Int J Telemed Appl.

[109] Black AD, Car J, Pagliari C, Anandan C, Cresswell K, Bokun T (2011). The impact of eHealth on the quality and safety of health care: a systematic overview. PLoS Med.

[110] Coiera E, Magrabi F, Talmon J (2017). Engineering technology resilience through informatics safety science. J Am Med Inform Assoc.

[111] Tarzia L, Valpied J, Koziol-McLain J, Glass N, Hegarty K. Methodological and ethical challenges in a web-based randomized controlled trial of a domestic violence intervention. J Med Internet Res. 2017;19(3):640. 10.2196/jmir.7039 PMC5388827 28351830

[112] Rees K, Zweigenthal V, Joyner K (2014). Implementing intimate partner violence care in a rural sub-district of South Africa: a qualitative evaluation. Glob Health Action.

[113] Link T (1999). Beneath the surface. J Libr Adm.

[114] Coiera E, Ash J, Berg M (2016). The unintended consequences of health information technology revisited. Yearb Med Inform..

[115] Harrison MI, Koppel R, Bar-Lev S (2007). Unintended consequences of information Technologies in Health Care—an Interactive Sociotechnical Analysis. J Am Med Inform Assoc.

[116] Oschwald M, Renker P, Hughes RB, Arthur A, Powers LE, Curry MA (2009). Development of an accessible audio computer-assisted self-interview (A-CASI) to screen for abuse and provide safety strategies for women with disabilities. J Interpers Violence..

[117] Robinson-Whelen S, Hughes RB, Powers LE, Oschwald M, Renker P, Swank PR (2010). Efficacy of a computerized abuse and safety assessment intervention for women with disabilities: a randomized controlled trial. Rehabil Psychol..

[118] Lin JD, Lin LP, Lin PY, Wu JL, Li CD, Kuo FY (2010). Domestic violence against people with disabilities: prevalence and trend analyses. Res Dev Disabil.

[119] Basile KC, Breiding MJ, Smith SG (2016). Disability and risk of recent sexual violence in the United States. Am J Public Health.

[120] Bonomi A, Nichols E, Kammes R, Green T (2018). Sexual violence and intimate partner violence in college women with a mental health and/or behavior disability. J Women's Health (Larchmt).

[121] Chan KL, Emery CR, Ip P (2016). Children with disability are more at risk of violence victimization: evidence from a study of school-aged Chinese children. J Interpers Violence..

[122] Neille J, Penn C (2015). The Interface between violence, disability, and poverty: stories from a developing country. J Interpers Violence..

[123] Salwen JK, Gray A, Mona LR (2016). Personal assistance, disability, and intimate partner violence: a guide for healthcare providers. Rehabil Psychol.

[124] Renblad K (2003). How do people with intellectual disabilities think about empowerment and information and communication technology (ICT)?. Int J Rehabil Res.

[125] El Morr C: Health Services Coordination: the role of virtual community. In: eHealth 2018 Conference, May 27-30, 2018. Vancouver; 2018.

[126] Bender JL, Jimenez-Marroquin MC, Ferris LE, Katz J, Jadad AR (2013). Online communities for breast cancer survivors: a review and analysis of their characteristics and levels of use. Support Care Cancer.

[127] El Morr C (2007). Mobile virtual communities in healthcare: managed self care on the move.

[128] El Morr C, Kawash J (2007). Mobile virtual communities research: a synthesis of current trends and a look at future perspectives. Int J Web Based Communities.

[129] Johnston AC, Worrell JL, Gangi PMD, Wasko M (2013). Online health communities. Inf Technol People.

[130] Chorbev I, Sotirovska M, Mihajlov D (2011). Virtual communities for diabetes chronic disease healthcare. Int J Telemed Appl.

[131] Vasconcellos-Silva PR, Carvalho D, Lucena C (2013). Word frequency and content analysis approach to identify demand patterns in a virtual Community of Carriers of hepatitis C. Interact J Med Res.

[132] Matura LA, McDonough A, Aglietti LM, Herzog JL, Gallant KA (2013). A virtual community: concerns of patients with pulmonary hypertension. Clin Nurs Res.

[133] El Morr C, Cole C, Perl J (2014). A health virtual Community for Patients with chronic kidney disease. Procedia Comput Sci.

[134] Frost J, Vermeulen EI, Beekers N (2014). Anonymity versus privacy: selective information sharing in online cancer communities. J Med Internet Res.

[135] Barry MJ, Edgman-Levitan S (2012). Shared decision making — the pinnacle of patient-centered care. N Engl J Med.

[136] Hansson E, Tuck A, Lurie S, McKenzie K, for the Task Group of the Services Systems Advisory Committee Mental Health Commission of Canada. Improving mental health services for immigrant, refugee, ethno - cultural and racialized groups issues and options for service improvement. Toronto: Mental Health Commission of Canada and the Centre for Addiction and Mental Health; 2010. p. 2018.

[137] Elwyn G, Frosch D, Volandes AE, Edwards A, Montori VM (2010). Investing in deliberation: a definition and classification of decision support interventions for people facing difficult health decisions. Med Decis Mak.

[138] Heidelberger CA, El-Gayar O, Sarnikar S (2011). Online health social networks and patient health decision behavior: a research agenda.

[139] Loane SS, D'Alessandro S (2013). Communication that changes lives: social support within an online health community for ALS. Commun Q.

[140] Short LM, Surprenant ZJ, Harris JM (2006). A community-based trial of an online intimate partner violence CME program. Am J Prev Med.

[141] Brock D, Kim S, Palmer O, Gallagher T, Holmboe E (2013). Usability testing for the rest of us: the application of discount usability principles in the development of an online communications assessment application. Teach Learn Med.

[142] Chamorro-Koc M, Popovic V, Emmison M (2009). Human experience and product usability: principles to assist the design of user-product interactions. Appl Ergon.

[143] Hegarty K, Tarzia L, Murray E, Valpied J, Humphreys C, Taft A, et al. Protocol for a randomised controlled trial of a web-based healthy relationship tool and safety decision aid for women experiencing domestic violence (I-DECIDE). BMC Public Health. 2015;15(1):736. 10.1186/s12889-015-2072-z PMC4522060 26231225

